# The prevalence, course, and risk factors of suicidal ideation and suicide attempts among students in vocational education

**DOI:** 10.1186/s13034-024-00828-7

**Published:** 2024-10-17

**Authors:** Milou Looijmans, Paula von Spreckelsen, Guus Berkelmans, Arne Popma, Diana van Bergen, Renske Gilissen, Saskia Mérelle

**Affiliations:** 1 Research Department, 113 Suicide Prevention, Amsterdam, The Netherlands; 2 Psychiatry, Amsterdam UMC, Amsterdam, The Netherlands; 3 Faculty of Pedagogical and Educational Sciences, University of Groningen, Groningen, The Netherlands

**Keywords:** Suicide prevention, Vocational education, Risk factors, Mental health, Students

## Abstract

**Background:**

Worldwide, suicide is one of the leading causes of death among adolescents and young adults. Given that suicide in this age group is common within vocational students, this study aims to provide insights into the prevalence, course, and risk factors of suicidal ideation (SI) and suicide attempts (SA) among students in vocational education over the past 10 years.

**Methods:**

This study has a repeated cross-sectional design, utilizing data from 2013 to 2023 provided by the ‘Testjeleefstijl’ foundation in the Netherlands (‘Test Your Lifestyle’). In total, 101,182 students in vocational education completed a web-based standardized questionnaire. Univariate logistic regression was used to test the predictive value of risk factors separately (anxiety and depression, gender, age and school year) on SI and SA. In addition, a machine learning model (Berkelmans et al., 2023) ​was used for high-risk identification of combined risk factors (multivariate models).

**Results:**

Within vocational students, 12-month SI and SA prevalence increased from respectively 17.7% and 2.3% in schoolyear 2013–2014 to 23% and 3.2% in 2022–2023. Although female gender significantly predicted SI and SA in the univariate analyses, the multivariate models revealed that female gender decreased the likelihood of both SI (OR 0.9) and SA (OR 0.7). A high risk for anxiety and depression was the strongest predictor in the multivariate models for SI (OR 42.8) and SA (OR 19.0).

**Conclusion:**

Over the past decade, the prevalence of SI and SA increased in students in vocational education, with the risk of anxiety and depression being the strongest contributing factor. While females had a higher prevalence of anxiety and depression, the results suggest these conditions tend to lead to SI and SA more quickly among male students. Early intervention in suicide prevention is crucial, highlighting the need to identify and address anxiety and depression. Vocational education schools have a critical role in this, emphasizing early screening and intervention, with specific attention to gender-specific factors.

## Introduction

Worldwide, suicide is a major public health problem. In fact, it is one of the leading causes of death among individuals aged 10 to 30 years [1]. In the Netherlands, where this study was conducted, suicide is the most common cause of death among young people aged 10 to 30 [2].

In general, the mental health of college and university students is monitored more frequently than that of students in vocational education [3–5]. However, a recent Dutch study that explored the characteristics of young adults who died by suicide in 2021–2022 revealed that young students who dropped out of vocational education prematurely were at a heightened risk for suicidal ideation (SI) [6]. Internationally, research on the link between education tracks and SI is limited. There are some indications suggesting an association between lower entry levels and increased SI [7–9]. SI is a risk factor and indicator for the early detection of both suicide attempts (SA) and fatal suicides [10]. So, to ensure inclusivity regarding risk factors for suicidal ideation and suicide attempts among young people from diverse educational backgrounds, it’s crucial to gain more insight in the prevalence and risk factors of students in vocational education.

Mental disorders, specifically affective disorders, are one of the strongest risk factors for youth suicide [11] but also for the development of SI as well as SA [12–14]. Especially during adolescence and early adulthood, depression may increase the propensity for engaging in risky behaviors, such as suicide attempts [12]. Moreover, depression has also been identified as a strong predictor of suicide risk in university and college students [15], being predictive of both SI and SA [16]. Other mental disorders such as anxiety disorders, which often co-occur with other mental health conditions (e.g., depression), can also elevate the likelihood of suicidal ideation, suicide attempts, or suicide [11]. For example, medical students with anxiety disorders seem to be more likely to show SI (OR = 3.02 [2.04–4.47] [16]). Consequently, with regard to this study, students in vocational education with a high risk on anxiety and depression may report more suicidal ideation and suicide attempts.

Next to psychological symptoms such as anxiety and depression, also demographic characteristics have been identified as risk factors to suicidality. With regard to gender, literature shows young men experience a suicide rate that is over two times higher than that of young women [17]. There is evidence indicating that female adolescents are more likely to experience SI and/or SA compared to male students [16, 18, 19]. Consequently, in this study, female students in vocational education may also represent a higher risk for suicidal ideation and suicide attempts.

Regarding age, the development from adolescence to adulthood presents a number of new challenges and difficulties for the still-developing brain, identity and personality of young adults which can increase the risk of SI and/or SA [20]. Some of these challenges refer to the struggles of starting to live an independent life and living on their own, such as (partially) losing the daily social support and financial security of living in the parental home and having to find new social connections. As 18 years of age often marks the time when young adults leave the house, with regard to our study, students in vocational education may especially be prone to report SI and/or SA around the age of 18 and a few years afterwards.

During the Covid-pandemic, (school years 2019–2020/2020–2021) the amount of Dutch young adults who died by suicide has increased by 15% [21]. During recent school years, a general decrease in mental health is observed in young people in the Netherlands [22]. In addition, in a large US cohort study, rates of major depressive disorder and suicidality strongly increased over a period of 7–8 years (2018/2019 − 2017) in young adults [23]. Furthermore, in 2022, the third annual mental state of the world report [24] found that young adults were particularly vulnerable to mental health problems. Based on this, in our study, vocational students in more recent school years may have a stronger association with SI and/or SA than students in less recent school years.

The main aim of the current study is to explore the prevalence, course, and risk factors (risk of anxiety and depression, gender, age, school year) associated with suicidal ideation and suicide attempts among students in vocational education in the Netherlands. We will explore the predictive value of the included risk factors associated with SI and SA in students over the past 10 years. In addition, we will use machine learning models to explore interactions of the included risk factors. This will allow us to examine whether subgroups that are at a particularly high risk for SI and SA can be identified. In sum, this study will not only contribute to the current knowledge about SI and SA among students in vocational education but will also offer important information for educational institutions and other organizations regarding high risk subgroups, promoting the implementation of preventive measures.

## Method

### Context of the Dutch educational system

The Dutch education system broadly encompasses primary education for all children from age 4 to approximately 12. Afterward, they move on to secondary education, where they pursue pre-vocational tracks (age 12–16) or pre-university tracks (age 12–17/18). Following this stage, they may proceed to vocational education (MBO; age 16+), higher professional education (HBO; age 17+), or university (age 18+). The current study focuses on students in vocational education (age 16+).

### Data and design

The data for this research came from the Dutch foundation ‘Testjeleefstijl’ (translation: ‘Test your lifestyle’) which collects data about students in vocational education about several lifestyle topics (e.g. physical & mental health, alcohol, drugs, sexuality) that concern young people between the ages of 12 and 25. Testjeleefstijl enables vocational education schools to have their students complete various digital questionnaires on a broad range of topics. Schools can access the results via a dashboard and have the ability to enable or disable certain topics, thus creating a customized combination of information for their own school. Due to privacy regulations, scores from the different topics cannot be linked to each other. As such, we were only able to utilize data pertaining to one topic (depression and suicidality) for this study. The data had a repeated cross-sectional design, which means that each year (from 2013 to 2023), a different group of students filled in the questionnaire.

### Participants and recruitment

The participants who filled out the questionnaires are students in vocational education, who completed a web-based standardized questionnaire between 2013 and 2023. The recruitment of participants was carried out by schools with a Testjeleefstijl subscription. Interested schools can register on the website and decide which questionnaires they want to administer to their students. Schools can also choose to have students complete the questionnaires at school or allow them to do so at home. The Testjeleefstijl data cannot be traced back to individual persons, and teachers therefore do not know the answers individual students provided in the questionnaires. Students were asked to provide informed consent while filling out the questionnaires on the website. Testjeleefstijl processes the answers by theme, and all data are stored anonymously and are therefore not traceable to personal student accounts. Completing the Testjeleefstijl questions on the topic ‘suicidality and depression’ is estimated to have taken approximately five minutes. Students did not receive any financial or other compensation for completing the questionnaires. The study was approved by the medical ethical committee of the Amsterdam University Medical Center (file number: 2023.0568).

### Measures

The current study included the following repeated cross-sectional data from the past 10 school years (2013–2023): suicide attempt, suicidal ideation, (risk of) anxiety and depression, gender, age and school year.

#### Suicidal ideation and attempts

Suicidal ideation was assessed with the question: ‘Have you ever seriously thought about ending your life in the last 12 months?’ with the answer options ‘Never/occasionally/sometimes/often’. For the analyses, the answers on the SI question were dichotomized into ‘yes’ (occasionally/intermittently/often) and ‘no’ (never). Suicide attempts were assessed with the question: ‘Have you tried to end your life in the last 12 months?’ and the answer options ‘Yes/No/No answer’. The ‘no answer’ scores were coded as missing values.

#### Risk of anxiety and depression

Anxiety and depression were assessed with the Dutch version of the Kessler Psychological Distress Scale (K10), a questionnaire with 10 questions about anxiety and depression [25]. These items ask to indicate how often (1 = never to 5 = always) a participant has felt, for example, very tired, nervous, hopeless, restless, or worthless in the past 4 weeks. Based on the answers, scores are calculated that indicate a low (scores of 10–15), moderate [16–29], or high (30–50) risk of anxiety or depression [22].

#### Demographic variables

Gender was assessed by a multiple-choice item with the options: male, female, and other. The option ‘other’ was added in 2020–2021. Because of this, the number of students who fall in this gender group was too small (0.2%) to include them in the analyses and were therefore excluded. Age was assessed by asking students to indicate their age. From 2019 to 2022, a drop-down menu was used with the following options: 15, 16, 17, 18, 19, 20, 21, 22, 23 and older. Age data before 2019–2022 with values below 15 or above 23 was coded as “15” or “23 and older”, respectively. For the analyses, age was categorized into three age groups: 15–17; 18–20; 21–23 and older.

School year was extracted based on the timestamp of each dataset, with the following values: 2013–2014; 2014–2015; 2015–2016; 2016–2017; 2017–2018; 2018–2019; 2019–2020; 2020–2021; 2021–2022; 2022–2023. For the analyses, school year was dichotomized into (school) years before corona (pre-corona: school years 2013/2014 until 2018/2019) and years during/past corona (corona: school years 2019/2020 until 2022/2023).

### Statistical analysis

Only students with complete data on the variables of the main analyses were included in analyses. Descriptive analyses were used to calculate the prevalence of SI and SA over time and the prevalence of different subgroups of gender, age, school year and risk of anxiety and depression. Univariate logistic regression analyses were performed to test the predictive value of the different risk factors, namely gender (female/male), age (15–17/18–20/21–23), anxiety and depression (low/moderate/high) and school year (pre-corona and corona) separately, per SI (yes/no) and SA (yes/no) as the outcome.

For the high-risk identification of suicidal ideation and suicide attempts the machine learning model developed by Berkelmans et al. [26] was used in Python. This model allows for the exploration of complex interactions of socio-demographic risk factors while maintaining interpretability [26]. The interaction features of the SI and SA models consisted of combinations of risk factors (see above).

#### Machine-learning model

A heuristic algorithm was devised to obtain interacting features which provide additional risk of suicide attempts or suicidal ideation or reduce these risks. The interaction features were prioritized based on statistical significance and model improvement. The algorithm consisted of three phases (for each model).

#### Preparation

The data was split into 2 datasets: the training set (50%) and the validation set (50%). The training set was used to find interactions of interest. This set was further divided into a primary training set (80% of the training set), and a control set (20% of the training set). The validation set was used to estimate the final model.

#### Phase 1: finding interactions of interest

We started with a base logistic regression model which included just the basic predictors. Interactions of interest to the model were then iteratively added. An empty list (L) was initialized to track all interactions that have been considered. This means that all possible combinations of the variables (gender, age, school years, risk of anxiety, and depression) were tested and recorded in the subsequent step.

#### Iterative step

We scored potential interactions of interest (that were no yet in the list L) based on the primary training set. If the interaction with the highest score was above a certain minimal threshold (T) we added it to the model as well as to the list (L). If there were no interactions with a score higher than T we moved on to phase 2. It was then evaluated whether this model actually improved performance on the control set when it was compared to the previous model that didn’t contain this interaction. If the model did not improve: we returned to the model without this interaction, and checked whether there were interactions between predictors of the model that were not in the list L. If there were interactions, the iterative step started again. If there were not, we moved on to phase 2.

#### Phase 2: estimating final model

To get unbiased estimates that are unaffected by multiple testing the final model was estimated on a separate dataset: the validation set. This allows for interpreting parameters and confidence intervals in the usual manner.

## Results

### Population characteristics and prevalence of suicidal ideation and attempts

In total 101,182 students in vocational education were included in analysis, 44,470 of them were men and 54,656 were women. See Table 1 for the participant characteristics. 59,080 students were between 15 and 17 years old, 29,711 students between 18 and 20 years old and 10,548 students were 21 years or older. Out of the 101,182 students who participated in the study over the past 10 years, 18.8% (19022) seriously considered ending their life ‘at least once’ to ‘quite often’ in the past 12 months and 2.8% (2833) of students actually attempted suicide in the past 12 months.

**Fig. 1:**
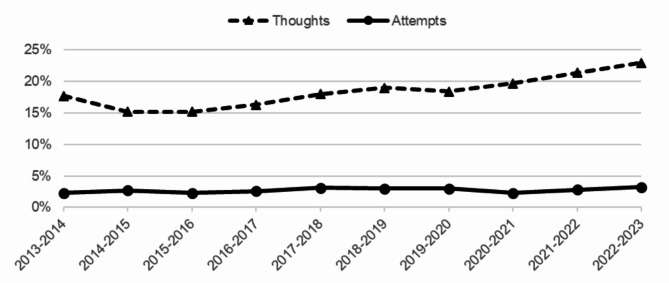
Prevalence of suicidal ideation and suicide attempts among students in vocational education from school year 2013/2014 until school year 2022/2023

Figure 1 displays the percentages of suicidal ideation and suicide attempts among students in vocational education over the past 10 years. In the school year 2013–2014, 17.7% of the students experienced suicidal ideation and 2.3% had attempted suicide in the past year. In the school year 2022–2023, 23.0% of the students experienced suicidal ideation and 3.2% had attempted suicide in the past year.

### Characteristics of the anxiety and depression risk groups

**Fig. 2:**
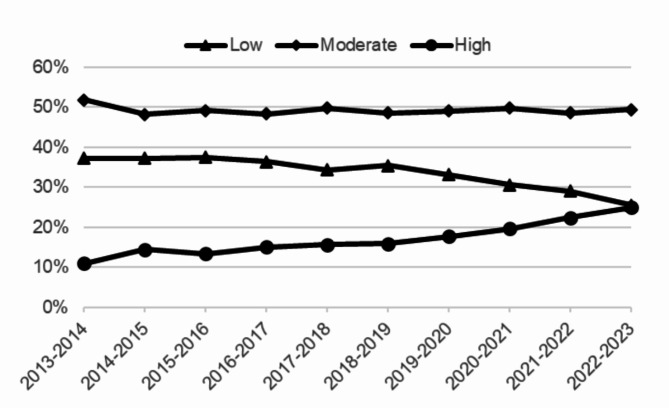
Prevalence of risk (low/moderate/high) of anxiety and depression among students in vocational education from school year 2013/2014 until school year 2022/2023

Table 1 presents the percentages of students in the low, moderate and high risk groups across the gender and age groups. Figure 2 presents the percentage of students in the anxiety and depression risk groups over the past 10 years. The 12-month prevalence of students scoring high on anxiety and depression was 10.9% in 2013–2014 and 24.9% in 2023–2024.

**Table 1:** Percentage of students in vocational education in the low, moderate and high anxiety and depression groups across gender and age

		Low risk	Moderate risk	High risk
Gender				
	Male	43.14%	45.32%	11.50%
	Female	24.40%	52.79%	22.77%
Age				
	15–17	34.59%	48.83%	16.53%
	18–20	30.09%	49.91%	19.99%
	21–23	29.84%	51.00%	19.14%

### Risk factors of suicidal ideation and suicide attempts

#### Gender

On average, 15.5% of all male students and 20.9% of all female students reported thinking about suicide at least once to quite often and 2.6% of male students and 2.8% of female students attempted suicide in the last year. Gender was a significant predictor of both suicidal ideation and suicide attempts in the univariate logistic regression analyses, with female students being 1.4 times more likely to experience suicidal ideation and 1.1 times more likely to have attempted suicide (see Table 2). When taking into account other risk factors in the multivariate machine learning model, female students were 0.8 times less likely to experience suicidal ideation (see Table 3) and 0.7 times less likely to have attempted suicide (see Table 3) in comparison with male students.

**Table 2:** Univariate Logistic Regression models

		*N*	Suicidal ideation		Suicide attempts	
			n (%)	Odds ratio (95% CI)	n (%)	Odds ratio (95% CI)
Gender						
	Male [ref.]	44,470	6878 (15.47%)	-	1153 (2.59%)	-
	Female	54,656	11,440 (20.93%)	1.44* (1.40–1.49)	1545 (2.83%)	1.11* (1.02–1.20)
Age						
	15–17 [ref.]	59,080	10,481 (17.74%)	-	1471 (2.49%)	-
	18–20	29,711	6024 (20.34%)	1.19* (1.15–1.23)	887 (2.98%)	1.22* (1.11–1.33)
	21–23	10,548	1957 (18.55%)	1.06 (1.00-1.12)	369 (3.50%)	1.42* (1.26–1.60)
School year						
	2013/2014–2018/2019 [ref.]	45,420	7576 (16.68%)	-	1193 (2.63%)	-
	2019/2020–2022/2023	55,762	11,312 (20.29%)	1.28* (1.25–1.33)	1576 (2.83%)	1.11* (1.03–1.20)
Risk anxiety/depression						
	Low [ref.]	33,148	890 (2.68%)	-	278 (0.84%)	-
	Moderate	49,889	7441 (14.92%)	6.37* (5.94–6.84)	788 (1.58%)	1.94* (1.69–2.23)
	High	18,103	10,553 (58.29%)	51.78* (48.17–55.74%)	1703 (9.41%)	14.67* (12.94–16.73%)

Note. * indicates p-value was smaller than Bonferroni-adjusted alpha value [adjusted *α*’s: gender & school year 0.05/2 = 0.025; age & risk for anxiety and depression: 0.05/3 = 0.017]

**Table 3:** Results logistic regression on validation sets suicidal ideation and suicide attempts

Suicidal ideation					Suicide attempts				
	N	Estimates	T-tests	OR (95% CI)		N	Estimates	T-tests	OR (95% CI)
B0 / Full population	9143	-3.52	-66.61	0.03 (0.03, 0.03)	B0 / Full population	1348	-4.66	-36.26	0.01 (0.01, 0.01)
Male	3466	0.00	0	1 (1, 1)	Male	580	0	0	1 (1, 1)
< 18	5199	0.00	0	1 (1, 1)	< 18	735	0	0	1 (1, 1)
Low risk anxiety/depression	455	0.00	0	1 (1, 1)	Low risk anxiety/depression	138	0	0	1 (1, 1)
2013/2014–2018/2019	3741	0.00	0	1 (1, 1)	2013/2014–2018/2019	607	0	0	1 (1, 1)
**Female**	**5677**	**-0.15**	**-4.38**	**0.86 (0.81**,** 0.92)**	**Female**	**768**	**-0.35**	**-5.29**	**0.7 (0.62**,** 0.80)**
**18–20**	**2970**	**0.13**	**3.74**	**1.14 (1.07**,** 1.23)**	18–20	430	0.22	0.36	1.02 (0.90, 1.16)
21+	974	0.05	0.96	1.05 (0.95, 1.15)	**21+**	**183**	**0.26**	**2.32**	**1.3 (1.04**,** 1.6)**
**Moderate risk anxiety/depression**	**3666**	**1.83**	**35.72**	**6.24 (5.64**,** 6.90)**	**Moderate risk anxiety/depression**	**367**	**0.80**	**5.71**	**2.23 (1.69**,** 2.94)**
**High risk anxiety/depression**	**5020**	**3.76**	**56.04**	**42.8 (37.53**,** 48.81)**	**High risk anxiety/depression**	**843**	**2.95**	**20.45**	**19.01 (14.34**,** 25.22)**
School year 2019/2020–2022/2023	5402	-0.01	-0.19	0.99 (0.94, 1.05)	**School year 2019/2020–2022/2023**	**741**	**-0.23**	**-3.17**	**0.8 (0.69**,** 0.92)**
**< 18 + High risk anxiety/depression**	**2856**	**0.24**	**4.35**	**1.27 (1.14**,** 1.42)**	Low risk anxiety/depression + 21+	19	0.29	1.09	1.34 (0.8, 2.25)
Male + High risk anxiety/depression	1549	0.06	1.06	1.06 (0.95, 1.20)	Low risk anxiety/depression + 2019/2020–2022/2023	68	0.26	1.23	1.25 (0.87, 1.8)
					Male + 2013/2014–2018/2019 + 21+	32	-0.07	-0.26	0.93 (0.56, 1.56)
					Male + 2013/2014–2018/2019 + high risk anxiety/depression	124	0.04	0.34	1.05 (0.81, 1.35)
					2013/2014–2018/2019 + 21+	72	-0.16	0.20	0.86 (0.57, 1.28)

#### Age

Table 2 presents the results of the univariate logistic regression analyses of age on suicidal ideation and suicide attempts. In comparison to students in vocational education aged between 15 and 17 years (17.7%), students aged between 18 and 20 years were 1.2 times more likely to experience suicidal ideation (20.2%). Students aged 21–23 or older were not found to have a significantly higher percentage of suicidal ideation (18.6%) than the lowest age group. With regard to suicide attempts, students aged between 18 and 20 years (2.9%) and between 21 and 23 years (2.5%) were 1.2 and 1.4 times more likely, respectively, to have attempted suicide in the previous year compared to students aged between 15 and 17 (2.5%). In the multivariate machine learning models (see Table 3), the age group of 18–20 remained a significant predictor of suicidal ideation (OR = 1.1) and the age group of 21–23 years remained a significant predictor of suicide attempts (OR = 1.3) when compared to the youngest age group (15–17 years).

#### School year

In comparison to the school years before 2019/2020, students were 1.3 times more likely to experience suicidal ideation and 1.1 times more likely to have attempted suicide in the school years from 2019/2022 to 2022/2023 (see Table 2 for the univariate logistic regression models). When taking into account other risk factors in the multivariate machine learning model, school year was no longer a significant predictor of suicidal ideation and the school years 2019/2022–2022/2023 were associated with a smaller likelihood (OR = 0.8) for suicide attempts compared to the school years before 2019/2022.

#### Risk for anxiety and depression

On average, 33.8% of the students had a low risk of anxiety or depression, 49.3% a moderate risk and 17.9% a high risk. In the univariate logistic regression models (Table 2), students with a moderate risk were 6.4 times more likely to experience suicidal ideation (14.9%) and 1.9 times more likely to have attempted suicide (1.6%) compared to students with a low risk. Students in the high risk group were 51.8 times more likely to experience suicidal ideation (58.3%) and 14.7 times more likely to have attempted suicide (9.4%) compared to students with a low risk. The moderate and high risk groups remained significant and strong predictors of both suicidal ideation and suicide attempts in the machine learning models (see Table 3).

#### Identification of high-risk groups for suicidal ideation and suicide attempts

Table 3 shows the results of the final machine-learning logistic regression models. All possible interactions have been tested, but only the interactions picked up by the training set are displayed in this table (see method). Examining interactions between risk factors of suicidal ideation, we can observe that students with the age 15–18 years and high anxiety and depression risk had a higher risk on suicidal ideation (OR = 1.3 (95% CI OR [1.1, 1.4])). No significant interactions between risk factors for suicide attempts were found.

## Discussion

This study was conducted to provide insight into the prevalence, course, and predictors of SI and SA among students in vocational education in the Netherlands in the past 10 years. Strikingly, an increasing trend can be observed over the past 10 years in both SI and SA, as well as in anxiety and depression risk. While in 2013, around 10% of vocational students were at a high risk for an anxiety or depressive disorder, in 2023 almost one fourth (25%) of vocational students fell into this high risk category. These results align with other global findings about a rising trend in mental health issues among adolescents [3, 27–31].

The univariate analyses indicated that female students in vocational education tend to engage in SI and SA slightly more frequently than males (OR SI = 1.4; OR SA = 1.1). However, when taking into consideration other risk factors, being female actually seems to offer some protection against SI (OR = 0.9) as well as SA (OR = 0.7). Possibly, the disparities in gender might stem from differences in the risk of anxiety and depression. Research worldwide consistently shows that the prevalence of anxiety and depression is two to three times higher in women compared to men, starting from puberty [32, 33]. Female students in vocational education experience a heightened risk of anxiety and depression but the progression toward SI and particularly SA, may occur at a slower rate compared to male students dealing with anxiety and depression. This corresponds with literature indicating that the duration of the suicidal process is much shorter in males than in females [34]. In addition, as women are more likely to engage in mental health help seeking, female students in vocational education with mental health concerns might be more likely to search and ask for help compared to male students [35].

Aligning with studies indicating that the transition from adolescence to adulthood is associated with several challenges and difficulties, such as developmental transitions, rapid sculpting of the brain, and the psychosocial tasks of forming social and personal identities, this study shows students aged 18–20 in vocational education are at a slightly higher risk (OR = 1.1) for SI. More importantly, in the age group under 18 years old, anxiety and depression are more likely to transition into SI compared to the age group above 18. This underscores the importance of early initiation of screening for anxiety and depression to mitigate the risk of transitioning to SI.

Just like other research indicating that the COVID-19 pandemic had a negative impact on the mental health of adolescents [36, 37], results from the univariate logistic regression analyses showed that the likelihood of SI and SA was somewhat higher in recent school years. However, the final machine learning model actually found that these years were associated with a slightly lower risk for SA. Looking at the course of anxiety and depression scores over the school years (Fig. 2), and the extremely high predictive value of moderate/high anxiety and depression risk scores to both SI and SA, this disparity might be explained by including anxiety and depression risk scores in the multivariate models.

Overall, anxiety and depression stand out as a particularly strong risk factor for both SI and SA. Students in the high risk group were around 40–50 times likelier to have experienced SI and around 15–20 time more likely to have attempted suicide compared to students in the low risk group. Age, gender, and pre/post-pandemic years were significant risk factors when examined on their own. However, in the combined models, only anxiety and depression risk scores stood out as strong risk factor to both SI and SA.

### Implementation recommendations

The current findings highlight the importance of a school-wide approach to tackle mental health concerns in vocational education. Regarding school-based prevention programs, there is increasing evidence for the effectiveness of reducing SI and SA among adolescents [38]. A recent meta-analysis by Walsh et al. (2023) highlighted the importance of expanding the focus of school-based suicide prevention and selected studies that measured the effect of school-based suicide prevention interventions with SI and SA as outcomes, focusing on three types of interventions: [1] addressing issues related to suicide to decrease its occurrence [2], integrating prevention efforts within broader mental health strategies, and [3] implementing proactive school-based suicide prevention interventions that indirectly lower SI and SA by focusing on risk and protective factors. The results showed a 13–15% reduction in SI and a 28–34% reduction in SA among 33,155 students in 329 schools [39].

Regarding gender differences, the results of the current study highlight, first and foremost, the importance of increased awareness of suicidal ideation (SI) and/or suicide attempts (SA) among men vulnerable to anxiety and depression. Secondly, this suggests the need for targeted screening and monitoring measures, particularly within vocational training programs predominantly attended by men, such as those in technical or construction fields. This is especially crucial since sectors with a high male workforce, such as industry, construction, and transportation, have relatively higher rates of suicide [40]. Proactive interventions for students at elevated risk of anxiety and depression are crucial.

### Strengths and limitations

The major strength of this study is that it provided more insight into the prevalence and risk factors of SI and SA among students in vocational education, a group about whom we know relatively little compared to college or university students, particularly in the Netherlands. In addition, because we used a heuristic machine learning method to detect interactions, our model had flexible settings which enabled us to fine-tune the balance between good model performance and statistically robust results. Also, the robustness and reliability of our findings are significantly enhanced by the size of the database with measurements over the past ten years. However, there were also limitations. First, this study utilized a repeated cross-sectional design, which involved collecting data from different individuals at multiple points in time. Due to the absence of a longitudinal design, we were unable to track changes within individuals over time, potentially restricting the depth of comprehension concerning temporal relationships and causal inference. Not being able to track students also prevented us to identified which students later dropped out of school, a group that has been identified at being at a particularly high risk for experiencing suicidal ideation [6]. On a methodological level, students who repeated a year and potentially filled in the questionnaires more than once, could not be identified and excluded from the analysis. As the amount of such students is expected to be negligible, we estimate the risk of potential bias to be low. Second, the assessment of gender did not specify whether it referred to the gender that a person identified with or sex (at birth) and individuals identifying as gender ‘other’ were excluded from the current analyses. This may have led to a lack of representation and potentially bias regarding non-cisgender individuals. Third, there were a restricted number of (non-demographic) risk factors considered in analysis since we used archived data. This implies that other potential influential factors (e.g., hopelessness, financial problems), may have been overlooked, thereby limiting the comprehensiveness and depth of the conclusions that were drawn. Fourth, a small part of the participants (4.8%) chose not to answer the questions about suicide SA (‘I do not want to answer this’). While we cannot draw definitive conclusions about the meaning of this response, this result may indicate an underreporting of the percentage of SA. Another limitation are the self-reporting questionnaires which could have potentially led to underreporting or overreporting of anxiety and depression, SI and SA.

We recommend future research to continue examining mental health and suicidality in the understudied population of vocational students. Based on the discussed limitations, we advise following students longitudinally to map out the temporal relationships between the course of suicidality and, next to depression and anxiety, important other factors in young adults generally (e.g., LHBTQ+, sleep disturbance, financial problems, loneliness, experiences of victimization, hopelessness [3, 15, 16, 41–43] and in vocational students specifically (e.g., school dropout [6]. This would allow us to gain a deeper understanding of the contribution of (the combination of) different factors on the course of suicidality and in turn provide us critical targets for intervention within the vocational school setting. Such an examination could for example provide insights into gender differences in the temporal process from suicidal ideation to behavior.

## Conclusion

Students in vocation education are an understudied group experiencing increasing rates of SI and SA. According to the findings, there has been a rise in both SI and SA over the past decade. However, a considerable part of this increase can be attributed to the heightened risk of anxiety and depression. Despite females exhibiting a greater prevalence of anxiety and depression, these conditions tend to precipitate SI/SA more rapidly among male students. Early intervention in suicide prevention is imperative, necessitating the identification and management of anxiety and depression, particularly within vocational education settings. Schools play a pivotal role in this endeavor, emphasizing the importance of early screening and intervention while also acknowledging gender-specific considerations.

## Data Availability

Data will be provided upon request.
